# *psbE-psbL* and *ndhA* Intron, the Promising Plastid DNA Barcode of *Fagopyrum*

**DOI:** 10.3390/ijms20143455

**Published:** 2019-07-14

**Authors:** Yue Huang, Zhiqiang Li, Chenglong Wang, Chenyan Zou, Wen Wen, Jirong Shao, Xuemei Zhu

**Affiliations:** 1 College of Life Sciences, Sichuan Agricultural University, Yaan 625014, China; 2 College of Environmental Sciences, Sichuan Agricultural University, Chengdu 611130, China; 3 Plateau Biological Resources R & D Platform of Xichen Corporation, National Agricultural High-tech Innovation Center, Chengdu 611130, China

**Keywords:** buckwheat, *Fagopyrum*, phylogenetic relationship, *psbE-psbL*, *ndhA* intron

## Abstract

Buckwheat is an important functional food material with high nutritional value. However, it is still a difficult task for the taxonomy studies of wild buckwheat that are only based on morphology. In order to demonstrate the most efficient DNA barcode in the phylogenetic research of buckwheat, promote the investigation of wild buckwheat, and also reveal the phylogenetic relationship between *Fagopyrum* species, *psbE-psbL* and *ndhA* intron were validated here, which previously have been proved to be promising DNA barcode candidates for phylogenetic studies in genera *Fagopyrum*. Meanwhile, *ndhA* intron + *psbE-psbL* and *matK* + *psbE-psbL* could distinguish the relationship between species clearly. Combining the results of morphology and molecular markers, we suggested the buckwheat species should be divided into two subgroups, one subgroup consisted of *F. tataricum*, *F. esculentum*, *F. cymosum* and its related wild species, and the other subgroup included other wild buckwheat species. Our results could fulfill molecular markers of taxonomy research in genera *Fagopyrum*, promote wild buckwheat species identification, and assist in the use of wild buckwheat resources in the future. Additionally, the phylogenetic relationship revealed here could provide valuable information for molecular breeding of buckwheat and provide reference for inter-species hybridization.

## 1. Introduction

Buckwheat is a nutritional and economically cope adapted to harsh environments, which belongs to the genera *Fagopyrum*. It has been widely distributed around the world and already praised as a potential functional food for tea, cookies, noodles and so on. Meanwhile, buckwheat contains high-quality proteins with a high content of essential amino acids; retrograded starch; multiple mineral elements; and abundant secondary metabolic products such as flavonoids, phenolic derivatives, and fagopyrin, which are recognized as the major bioactive components for heath improvement and disease treatment [1,2]. At the same time, the protein in buckwheat is gluten-free, and it will process buckwheat and its products as an alternative nutritious food to substitute the gluten grains without causing allergens and digestive issues [3,4]. Subsequently, buckwheat contains rare bioactive components such as rutin, quercetin, vitexin, anthocyanidins, and myo-inositol, which play an important role in anti-oxidation metabolism of the human body as scavengers of active oxygen and possess healing effects on some chronic diseases like diabetes [5], fatty liver [6], and even cancer [7]. Additionally, it is should be noticed that buckwheat is the only pseudocereal rich in natural rutin, which process buckwheat became a beneficial source of dietary rutin [2]. Consequently, buckwheat and its food products have been paid more and more attention to due to its valuable bioactive compounds. Meanwhile, buckwheat has also been treated as a substitute for main food especially in high mountainous areas like the Himalayan region, and it has already become a common food in Southwest China, as well as other regions in East Asia, Europe, and North America [8]. Southwest China is the original birthplace of buckwheat after morphological, cytology and molecular researches due to its complex geographical environments and variable climatic characteristics [9,10,11]. Before the European scientists began to search for wild buckwheat species in China, there were only three buckwheat species, *F. esculentum*, *F. tataricum*, and *F. cymosum*, which were discovered at the end of the 19th century [12]. Nowadays, 26 buckwheat species of *Fagopyrum* have been confirmed and reported by botanists [13], most of which are wild buckwheat species, with three identified species *F. hailuogouense*, *F. luojishanense* and *F. longzhoushanense* [14]. On the other hand, molecular markers-based classifications are reliable in taxonomy and phylogenetic researches, and combined with morphological studies, all buckwheat species in genera *Fagopyrum* have been divided into two subdivisions, cymosum group and urophyllum group [9], and this result has been supported by phylogenetic research in recent years [15].

However, the phylogenetic relationship between different species in *Fagopyrum* still needs to be explored because of the insufficient and non-systematic plant materials and non-specificity molecular markers, which also make the results contradictory in different studies. For example, it was considered that *F. cymosum* seems more distantly related to *F. esculentum* in morphology and isozymes, but the molecular phylogenetic researches based on chloroplast genomes proved that *F. cymosum* has a close relationship with *F. tataricum* rather than *F. esculentum* [16]. Subsequently, many researchers have reported the phylogenetic relationship between species used different molecular markers, like *matK/trnK* [15,17], *FLO/LFY* [18], *rbcL-accD* [19], and so on. To elaborate the taxonomy status and the new species identification, however, some of these researches did not use outgroup species for the phylogenetic analysis [17,20], and the results are not comprehensive [16]. Normally, the phylogenetic relationship investigation based on nuclear genome sequence is different to that constructed by chloroplast genome information, which could suggest hybridization in the urophyllum group of *Fagopyrum* [18]. Some results regarding the phylogenetic relationship among buckwheat species is still contradictory and incongruence. The *F. qiangcai* is classified into the urophyllum group according to the fruit characterization, as well as this species should be classified into the cymosum group based on the cotyledons criterion which was proposed by Zhou [15]. Basically, the details of morphology data from different buckwheat could reflect the differences between species. However, it is still a difficult task to identify the wild buckwheat species or groups only based on morphological characters, because it is hard to find a key character to separate different species clearly, especially the transitional species [13]. Therefore, it will be very meaningful to the wild buckwheat investigation and the molecular breeding, through fully collecting wild buckwheat resources and using specificity molecular markers like *psbE-psbL* and *ndhA* intron, which have been reported after comprehensive comparative analysis based on the chloroplast genome of buckwheat [16] and have not been used in buckwheat phylogenetic research yet. Additionally, the previous research was only based on morphological characteristics and single molecular markers such as *RAPD*, *AFLP*, *matK* and so on, which did not provide enough evidence for the phylogenetic relationship verification [10,11,15,17,21]. It is also necessary to verify the potential of the utilization of *psbE-psbL* and *ndhA* intron in the research of the phylogenetic relationships of buckwheat. The research about *psbE-psbL* and *ndhA* intron could be treated as the useful extension from chloroplast genome research to phylogenetic analysis. On the other hand, it is still needed to reveal the phylogenetic relationship of the recently identified wild buckwheat species *F. luojishanense* and *F. longzhoushanense* in *Fagopyrum* [14], especially the taxonomy status of these two species. 

In this study, we will explore the phylogenetic relationships and taxonomy status of different buckwheat species in Southwest China through the use of multiple molecular markers including *psbE-psbL* and *ndhA* intron combined with morphological analysis results at the same time. After that, the potential of *psbE-psbL* and *ndhA* intron in phylogenetic research of buckwheat will be uncovered. Our research will provide richer molecular information that helps clearly distinguish the relationship among different buckwheat species and will make a further evaluation of different plastid DNA barcoding sequences in the molecular characterization of wild species and cultivated accessions of *Fagopyrum*. Afterwards, we would find more potential and credible genetic markers in buckwheat research.

## 2. Results

### 2.1. Analysis Based on Morphological Characteristics of Wild Buckwheat

**Figure 1:**
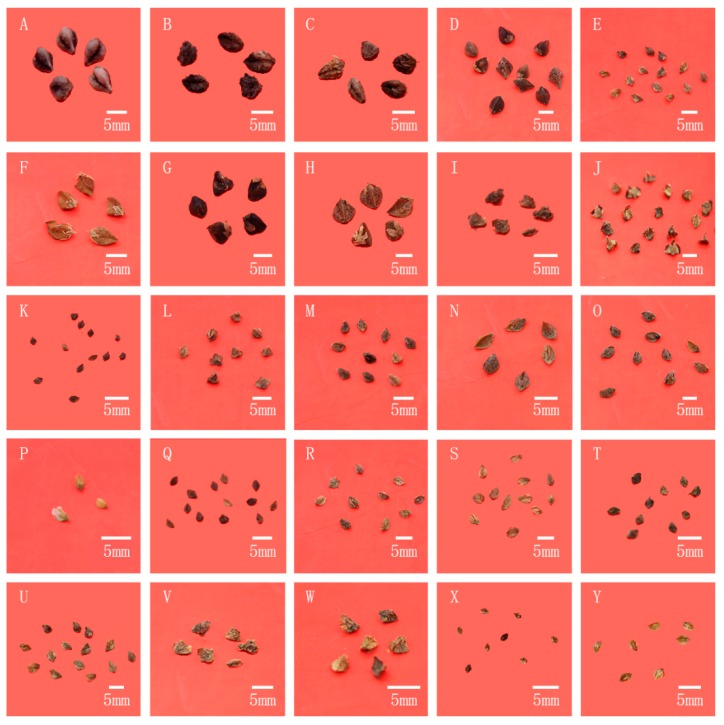
Fruit morphology of different species in *Fagopyrum*. Note: The species names of fruits (**A**–**Y**) as follows: *F. tataricum*, *F. tataricum* (sichuan), *F. tataricum* (yunnan), *F. esculentum* cv. *T12*, *F. esculentum* ssp. *ancestralis*, *F. megaspartanum*, *F.cymosum* (sichuan), *F.cymosum* (yunnan), *F. gracilipes* var. *odontopterum-R*, *F. luojishanense*, *F. jinshaense*, *F. longzhousahnense*, *F. rubifolium*, *F. qiangcai*, *F. callianthum*, *F. wenchuanense*, *F. capillatum*, *F. pugense*, *F. urophyllum*, *F. leptopodum*, *F. gracilipes*, *F. crispatifolium*, *F. gracilipes* var. *odontopterum*, *F. lineare*, *F. macrocarpum*.

As the edible part of buckwheat, the morphological characteristics of buckwheat grain are the most important index for the evaluation research of buckwheat. In this way, the fruits of different buckwheat species were observed and compared first. The fruit morphological details were shown in Figure 1. Based on the morphology of different buckwheat fruits, the differences among buckwheat species are easy to reveal. Subsequently, all the buckwheat in this research were divided into two parts. Species whose achenes length were longer than their perianths, were called the big achene group, and the other buckwheat species whose achenes length were almost equal to that of their perianths, were called the small achene group [22]. In the big achene group, the cultivated species and its related wild species collected from different locations are clearly distinguished with other buckwheat species, including *F. tataricum* and *F. esculentum* cv. *T12*, as well as *F. tataricum* (sichuan)*, F. tataricum* (yunnan)*, F. cymosum* (sichuan)*, F. cymosum* (yunnan) and *F. megaspartanum*; meanwhile, two wild buckwheat species could also be separated into this group based on the fruit morphology including *F. qiangcai* and *F. callianthum* with the average length of seeds being more than 4.5 mm. On the other hand, the small achene group was composed of mostly wild buckwheat species including *F. esculentum* ssp. *ancestralis, F. luojishanense; F. jinshaense, F. longzhoushanense, F. rubifolium, F. wenchuanense, F. capillatum, F. pugense, F. urophyllum, F. leptopodum, F. crispatifolium, F. lineare, F. gracilipes, F. gracilipes* var. *odontopterum* and *F. macrocarpum*; the average length of seeds was less than 4.5 mm, and most of them were less than 3.5 mm. The smallest seed in these buckwheat species was *F. jinshaense*, the average length of seeds was 2.1 mm and the average width of seeds was 1.5 mm, flow with *F. leptopodum* and *F. lineare*. All in all, it is also indicated that the relationship revealed in *Fagopyrum* is quite limited, which divided buckwheat species into several groups directly only based on morphological evidences, and the taxonomy results of individual wild buckwheat still needs to be described. 

**Figure 2:**
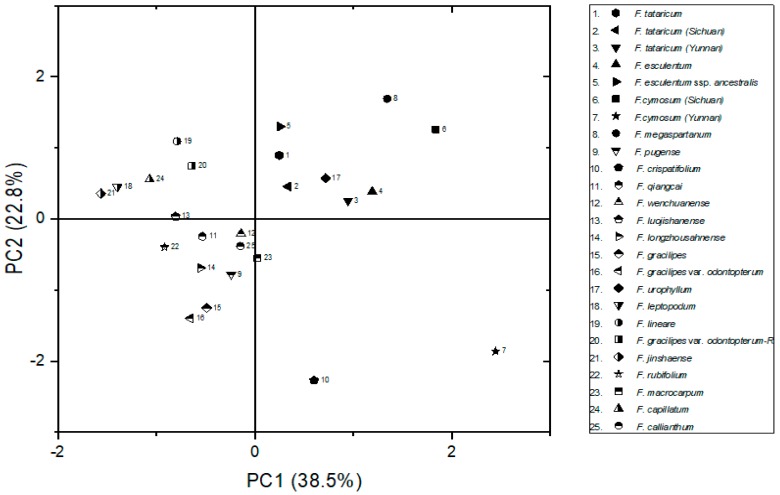
Scatter plot of *Fagopyrum* based on two main factors of PCA.

After that, the principal components analysis (PCA) was processed to reflect the differences among species in *Fagopyrum*, which will reduce the dimensionality of the morphology data from leaf, fruits, chromosome, karyotype, and reproductive patterns. The scatter plot drawn by two component factors (the details were showed in supplementary material 1) after PCA is illustrated in Figure 2. Based on the scatter plot, all buckwheat species in this research were separated into two parts. The *F. tataricum, F. tataricum* (sichuan)*, F. tataricum* (yunnan)*, F. esculentum* cv. *T12, F. esculentum* ssp. *ancestralis, F. cymosum* (sichuan)*, F. cymosum* (yunnan)*, F. megaspartanum,* and *F. urophyllum* clustered together; the other wild buckwheat also clustered together. Subsequently, *F. urophyllum* was quite distant to the other wild buckwheat, which means *F. urophyllum* has some similarities with *F. tataricum* and *F. esculentum* ssp. *ancestralis* in morphological characteristics, especially leaf, fruits and plant height. Interestingly, they were differing considerably in habit and gross morphology. Additionally, the *F. cymosum* (sichuan) and *F. megaspartanum* showed a closer relationship than other buckwheat. However, it was still different to the identified buckwheat in one component factor, for example, *F. longzhoushanense,* and *F. leptopodum* were the same in the horizontal component factor, both of them were 0.82. So it is necessary to evaluate the phylogenetic relationship using molecular markers.

### 2.2. psbE-psbL and ndhA Intron Are the Promising Molecular Markers in Fagopyrum 

**Figure 3:**
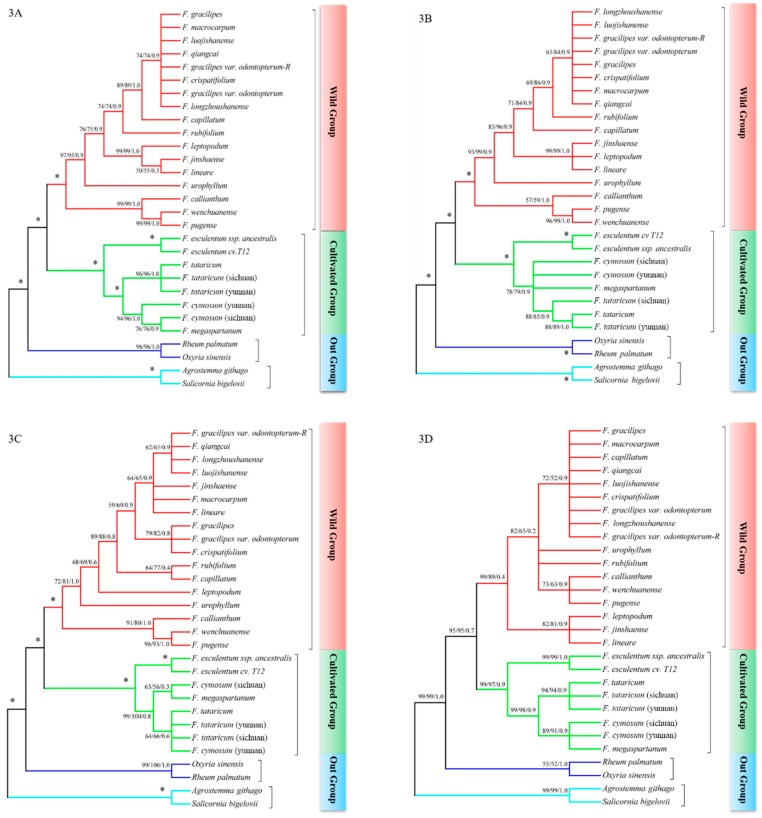

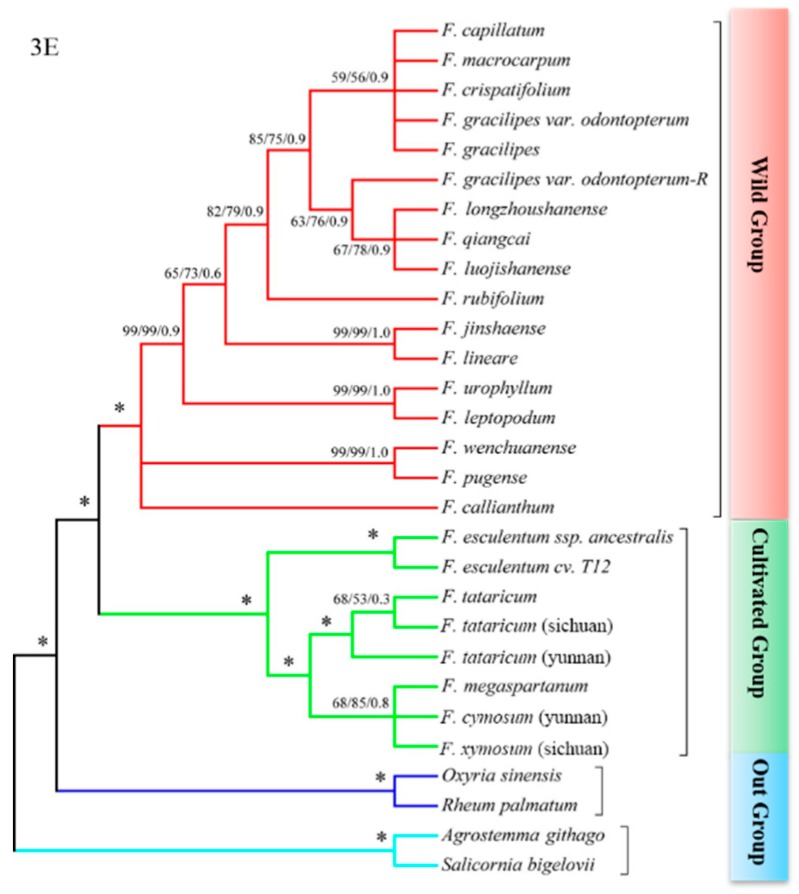
Phylogenetic analysis of genus Fagopyrum based on plastid DNA barcode. The figure (**A**–**E**) represent *matK*, *ndhA* intron, *rbcL-accD*, *trnT-trnL* and *psbE-psbL*, respectively. The * symbols in the phylogenetic tree show that the support rate of this branch is 100/100/1.0.

The phylogenetic trees constructed by the sequence information of *matK*, *rbcL-accD*, *trnT-trnL*, *psbE-psbL* and *ndhA* intron based on MP/ML/BI methods are shown in Figure 3. The primers designed for the phylogenetic analysis were showed in supplementary Table S1 of supplementary material 2, and the electrophoresis for PCR products for the *ndhA* intron could be found in supplementary Figure S1 of supplementary material 2. The out groups here came from Polygonaceae, Caryophyllaceae, and Chenopodiaceae, respectively, which could provide enough sequences information for phylogenetic analysis in *Fagopyrum*. Meanwhile, the phylogenetic trees in Figure 3 show the results of ML analysis; the trees illustrated are completely coincident with the other trees that were constructed based on MP and BI analysis. Subsequently, all the phylogenetic trees only show branches with bootstrap values more than 50, and the star symbols on the branches of phylogenetic trees represent the support rate which was 100/100/1.0. 

From our results, the phylogenetic trees built by different molecular markers showed similar topology structure, and it is also clear that all buckwheat species in this study belong to the same genera. Meanwhile, the outgroup came from different genera divided into two subgroups that colored blue and cyan of the branches, and all species from *Fagopyrum* were classified into one big group. Additionally, our results showed that all *Fagopyrum* species were separated into two subgroups with high internal resolution, which were colored red and green in Figure 3 and marked as wild buckwheat and cultivated buckwheat respectively. The cultivated buckwheat consisted of cultivated buckwheat and its related wild species, including *F. tataricum, F. esculentum, F. esculentum* ssp. *ancestralis, F. cymosum*, and *F. megaspartanum*.

On the other hand, the topology structure of phylogenetic trees built by *psbE-psbL* (Figure 3E) and *rbcL-accD* (Figure 3C) were more precise than others, which could reveal the relationship between transitional species with similar morphology clearly, such as the polygenetic relationship among *F. luojishanense*, *F. longzhoushanense, F. capillatum, F. crispatifolium, F. gracilipes, F. gracilipes* var. *odontopterum*, *F. qiangcai*, and *F. macrocarpum*, which could not be clearly revealed using the other molecular marker. Since the phylogenetic tree only showed the evolutionary branches with bootstrap values higher than 50, it indicated *psbE-psbL* could be used to investigate the phylogenetic relationship between the transitional species and morphologically similar species which used *matK* and other molecule markers which were hard to reveal. Compared with the other widely used marker *rbcL-accD*, the phylogenetic tree built by *ndhA* intron was better than that of *rbcL-accD*, which could reveal the phylogenetic relationship of *F. tataricum, F. esculentum* and *F. cymosum* precisely. Additionally, the *trnT-trnL* seems to not be good for the phylogenetic study of buckwheat compared with others due to the low bootstrap values.

### 2.3. psbE-psbL + ndhA Intron and psbE-psbL + matK Could Revealed the Relationship between Species Clearly

**Figure 4:**
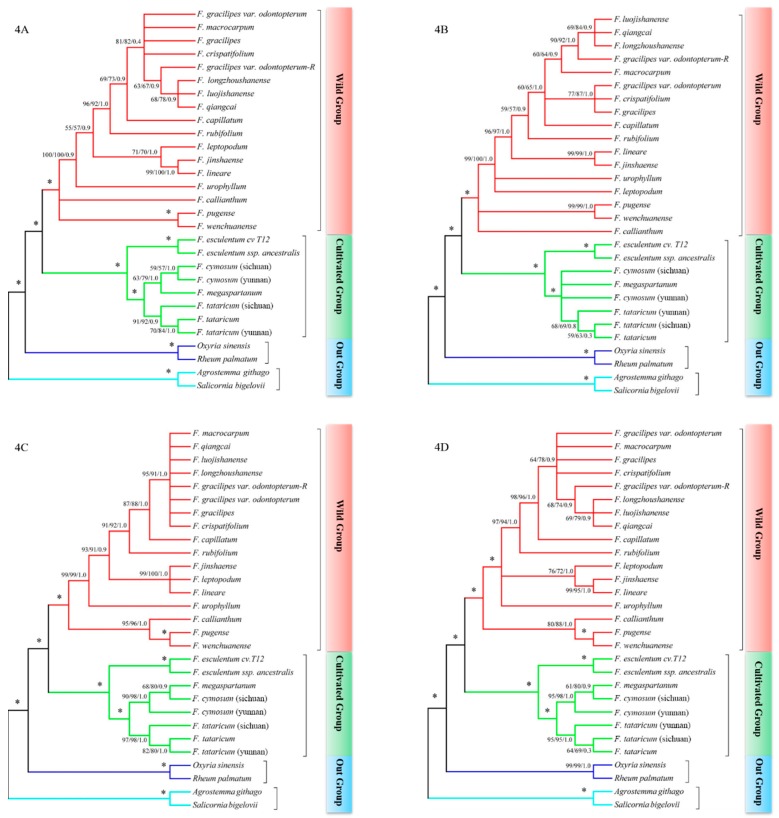

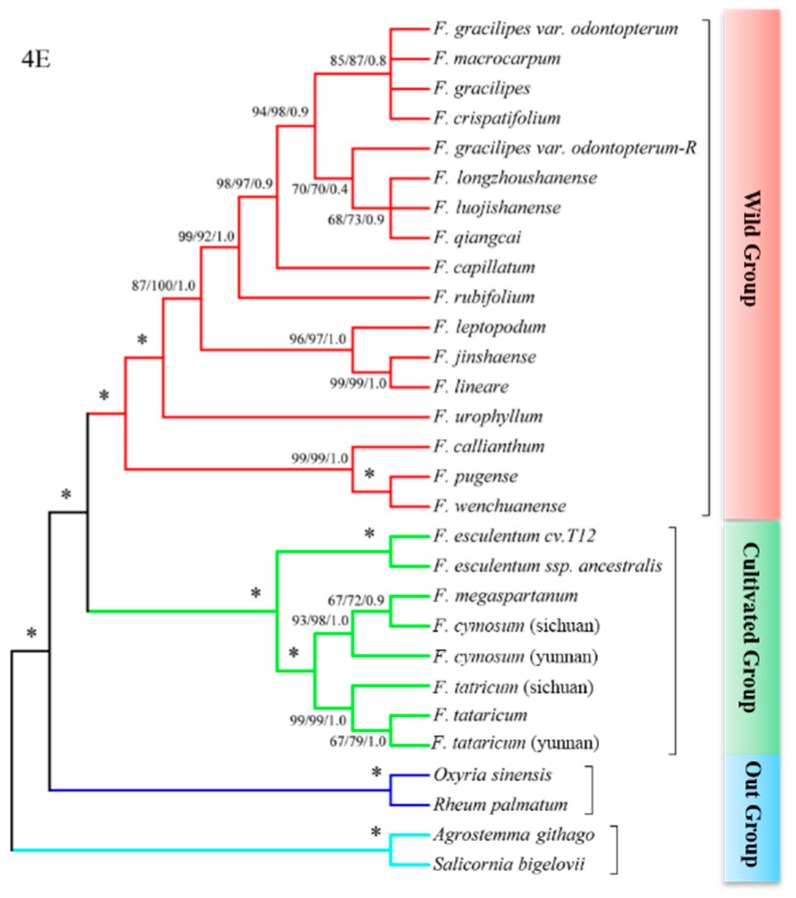
Phylogenetic analysis of genus Fagopyrum based on multiple combinations of DNA barcodes. The figure (**A**–**E**) represent *ndhA* intron + *psbE-psbL, rbcL-accD* + *psbE-psbL, matK + ndhA* intron, *matK* + *psbE-psbL,* and *matK* + *psbE-psbL+ ndhA* intron, respectively. The * symbols in the phylogenetic tree show that the support rate of this branch is 100/100/1.0.

From the phylogenetic trees in Figure 4, which illustrated ML trees coincident with the MP and BI methods, we only display the evolutionary branches with bootstrap values higher than 50, which means the low base substitution among species was ignored. All results showed a clear relationship between buckwheat but with higher bootstrap values than only using signal molecular markers, and the topological structure of phylogenetic trees built by three marks was the best out of all the combinations. The results showed all species in *Fagopyrum* clustered together and *Oxyria sinensis* and *Rheum palmatum* clustered together as the outgroup of *Polygonaceae*; meanwhile, *Agrostemma githago* and *Salicornia bigelovii* were divided into other outgroups that came from other sections. On the other hand, all buckwheat species were classified into two subgroups, a wild buckwheat group and cultivated group. In the cultivated group, *F. cymosum, F. tataricum* and *F. esculentum* were formed into three subgroups and the bootstrap values of these three subgroups were higher than 93, which was different to the other wild buckwheat group. Meanwhile, the *F. tataricum* and its related wild species consisted of one subgroup, as well as *F. esculentum* and its wild ancestors *F. esculentum* ssp. *ancestralis* clustering together, and *F. cymosum* clustering with *F. megaspartanum*, which demonstrated *F. megaspartanum* should be divided into *F. cymosum*. Additionally, the *F. cymosum* subgroup was closer to *F. tataricum* subgroup than the *F. esculentum* subgroup with the support rate of the branch being 100/100/1.0. In addition, these results also suggested that *F. cymosum* was more closely related to *F. tataricum* at the molecular level. At the same time, it was found that the relationship between *F. qiangcai, F. macrocarpum, F. crispatifolium,* and *F. gracilipes* still needed to be processed. 

Further, compared with other combinations based on two molecular markers, the phylogenetic trees built by *ndhA* + *psbE-psbL* and *matK* + *psbE-psbL* could reveal the relationship among species better than the other two. Due to the ambiguous topological structure in the cultivated group of the phylogenetic tree based on *rbcL-accD* + *psbE-psbL* and the unclear relationship between transitional species with similar morphology such as *F. luojishanense, F. longzhoushanense, F. crispatifolium, F. gracilipes, F. gracilipes* var. *odontopterum* and so on. Subsequently, the phylogenetic tree built by *psbE-psbL* + *ndhA* and *matK* + *psbE-psbL* further confirmed the reliability of the relationship between wild buckwheat species and the topological structure between two subgroups in *Fagopyrum*. More important is the fact that the topological structures and affinity among buckwheat species were basically the same with that which came from the phylogenetic tree based on three DNA barcodes, which are illustrated in Figure 4A,D,E.

Finally, all consistent phylogenetic trees constructed by multiple DNA barcodes speared buckwheat species into two big groups that had a high bootstrap value of 100, which also proves that the *psbE-psbL* and *ndhA* intron could be used as the ideal molecular markers for the study of the evolutionary relationship among *Fagopyrum*. Meanwhile, we suggested that the *ndhA* + *psbE-psbL* and *matK* + *psbE-psbL* could distinguish the relationship between buckwheat species reliably.

### 2.4. The Phylogenetic Relationship between Species in Fagopyrum 

To summarize our results for different molecular markers, we could demonstrate the relationship between species in *Fagopyrum*. Our results from phylogenic trees based on single and multiple DNA barcodes all indicated the *Fagopyrum* species should be divided into two groups, a wild buckwheat group and cultivated group, which have similar topology structures and stable bootstrap rates. The wild buckwheat group should consist mostly of wild species including *F. urophyllum, F. jinshaense, F. leptopodum, F. gracilipes, F. gracilipes* var. *odontopterum, F. wenchuanense, F. qiangcai, F. crispatifolium, F. rubifolium, F. callianthum, F. lineare, F. capillatum* and *F. macrocarpum*. Meanwhile, the cultivated group should contain *F. tartaricum, F. esculentum, F. cymosum* and its related wild species from different locations and also *F. megaspartanum* which we believe should be treated as *F. cymosum*. 

From our results, we inferred that *F. callianthum* is in a primitive position to the wild buckwheat group, and it clustered with *F. wenchuanense* and *F. pugense*, following with *F. urophyllum*. Meanwhile, *F. lineare, F. leptopodum,* and *F. jinshaense* have a relatively close relationship, as well as *F. qiangcai, F. luojishanense, F. longzhousahnense, F. gracilipes* var. *odontopterum-R, F. crispatifolium, F. gracilipes, F. gracilipes* var. *odontopterum* and *F. macrocarpum* with a close affinity. On the other hand, the cultivated buckwheat and its related wild species from different locations always gathered together, which reflected a small genetic divergence within cultivated species and its related wild species. Additionally, *F. megaspartanum* should be classified into *F. cymosum*, and our results also proved that the *F. cymosum* was more closely related to *F. tataricum* at the molecular level.

Further, more importantly, *psbE-psbL* could distinguish the wild buckwheat from cultivated buckwheat accurately during buckwheat resource investigations, as well as the evolutionary distinction between wild species, especially for wild species with similar morphology that cannot be distinguished clearly only by molecular markers such as *matK*. All in all, our research indicated that the *psbE-psbL* could further illustrate the relationship between buckwheat species similar to the *ndhA* intron.

## 3. Discussion

### 3.1. The Morphological Characteristics Are Not Enough for the Phylogenetic Study in Fagopyrum

At the end of 20th century to the beginning of 21st century, more and more buckwheat species were reported and identified by botanies through wild buckwheat resource investigation [9,14]. Genetically, the morphological characteristics are the basis of phylogenetic study. However, it also induces lot of synonym names in *Fagopyrum*, where 81 scientific plant names of species have ranked in The Plant List [23] because of similar morphological characteristics between transitional species. The principle for the investigation of new wild buckwheat is the identification of differences between species based on the morphological characteristics in fruit, flowers and leaves, which is mainly because it will be easier to reflect the evolutionary relationship between species by observing the morphology indicators directly than processing the nucleotide sequence information in the wild resource investigation. Sometimes, it is easy to distinguish wild species and cultivated species accurately only through morphological characters such as plant height, leaf and fruits. After research on the morphology of achene, DNA polymorphism and isozymes, Ohnishi demonstrated the species in *Fagopyrum* should be divided into a cymosum group and urophyllum group [9], which suggests that the cymosum group of buckwheat has greater dreary achene with a partial covered perianth, and the urophyllum group including specie with littler shiny achene. Meanwhile, the cymosum group consisted of *F. tataricum*, *F. esculentum* and *F. cymosum*, while other wild buckwheat should be classified as part of the urophyllum group. Chen studied the big-achene group (cymosum group reported by Ohnishi) of *Fagopyrum*, and he suggested this group should contain the *F. esculentum* subsp. *ancestrale, F. tataricum, F. homotropicum* and the other four *Fagopyrum* species [24,25].

In this research, in order to detect the morphological differences among species, which could reflect the genetic divergences in *Fagopyrum*, and to also fully understand the phylogenetic relationships between species, the morphological characteristics of individual buckwheat were observed and analyzed first. From the results of PCA, the cultivated species and its related wild buckwheat was separated from other wild buckwheat species. It also suggested that the wild buckwheat could be distinguished from cultivated buckwheat and its related wild species using multiple morphological characteristics. Based on our results of morphological characteristics, it indicated that the buckwheat should be divided into two groups which basically agree with the theory reported by Ohnishi [9]. Meanwhile, the cultivated species cluster together with its related wild species collected from different locations, which also indicated the genetic diversions were small between cultivated species and its wild buckwheat species. However, it still cannot well determine the distinction among transitional species of wild buckwheat clearly, and the relationship between some wild buckwheat species was still not clear enough due to some buckwheat overlapped in one principal component factor, especially the transitional species, such as *F. wenchuanense, F. capillatum,* and *F. callianthum*, which clustered very closely in the PCA result based on morphological characters. All in all, the phylogenetic relationships in *Fagopyrum* need further investigating, and only using morphological characteristics to reveal the relationship between buckwheat is not enough. Therefore, efficient molecular markers were required to demonstrate the phylogenetic relationship between species in *Fagopyrum*. 

### 3.2. The psbE-psbL and ndhA Intron Could Better Explore the Phylogenetic Relationship in Fagopyrum

Then, indeed, it is still difficult to find a feature to separate species completely in the one subgroup only based on morphological data. Moreover, there are many types of achene, and with a large change in fruit size, it is difficult to classify species directly. More importantly, there are still some transitional types which cannot be distinguished clearly. The DNA barcodes came from the high variable sequence which have been used as useful tools in phylogenetic research for many years. Hu et al. reported the *ndhF-rpl32* sequences could be used to distinguish *F. esculentum* ssp. *ancestralis* and *F. esculentum*, which indicated that the *ndhF-rpl32* was more effective in analyzing the phylogenetic relationships of buckwheat species [26]. Subsequently, the matK sequence has been widely used in the study of evolution and the phylogenetic relationship [27], as well as the *matK* and *rbcL-accD* has been reported for evolution study in buckwheat previously [27,28]. 

In order to verify the potential application of the molecular markers, *psbE-psbL* and *ndhA* intron, we also analyzed the phylogenetic relationship between different buckwheat. Five molecular markers were selected for the phylogenetic analysis, including the *psbE-psbL* and *trnT-trnL* intergenic region located in the large single copy (LSC) region of chloroplast and *ndhA* intron located in the small single copy (SSC) region of chloroplast, which came from the sequence divergence hotspot regions analysis of the chloroplast genome with the nucleotide diversity being higher than 0.06 performed by the DnaSP 5.0 software [16]. On the other hand, two molecular markers *matK* and *rbcL-accD*, which have been used in previous studies widely [14,15,17,19,27,28], were used to verify the results of the three high variation regions mentioned above. At the same time, we also used a single molecular marker and multiple molecular marker-combined information to construct the phylogenetic tree. The results will demonstrate the most efficient DNA barcode in the phylogenetic research of buckwheat, and also prove to be useful information for the species identification, taxonomy and genetic research in *Fagopyrum*, as well as for revealing the phylogenetic relationship between *Fagopyrum* species.

Our research results enrich the resource of DNA barcodes for phylogenetic study in *Fagopyrum* based on the results of comparative analysis of chloroplast genome, which has great advantages for selecting molecular markers based on chloroplast genome information and is used for evolution and relationship study, due to the abundant polymorphism and matrilineal inheritance [29]. Meanwhile, the *psbE-psbL* have been suggested as molecular markers for the phylogenetic research at low taxonomic levels [30,31,32], comparative genomics research [33], and cross-taxonomic surveys [34], but it has not been widely used previously. At the same time, *ndhA* intron seems to be especially variable in buckwheat [16], and both of them have not been used for phylogenetic relationships investigation in *Fagopyrum* yet. Our research proved the *psbE-psbL* and *ndhA* intron has great potential in interspecific relationships research of buckwheat. Based on our results, it was found that the promising plastid DNA barcode could elucidate the evolutionary relationship between different species of buckwheat clearly, especially for the wild buckwheat and cultivated buckwheat; therefore, *psbE-psbL* and *ndhA* intron have great potential for application in wild buckwheat species identification. Meanwhile, compared with the traditional molecular marker *matK* and *rbcL-accD*, the phylogenetic trees were constructed by *psbE-psbL* and *ndhA* intron with higher bootstrap values, which indicated they could be used as molecular markers for phylogenetic and taxonomic research of buckwheat. Additionally, the results of evolutionary analysis combined with multiple chloroplast regions are more reliable [35] and also proved the accuracy of our results. We demonstrated that the *matK* + *psbE-psbL, psbE-psbL* + *ndhA* intron two combinations could better distinguish the relationship between buckwheat species in detail. 

### 3.3. Reconstructing the Phylogenetic Relationship in Fagopyrum Consolidate by Multiple DNA Barcodes

Additionally, there are still many controversial problems in the phylogenetic study of buckwheat, and the main reasons for these problems are as follows: 1) First of all, it is mainly because the materials used before were not systematic and comprehensive. 2) Secondly, the morphological differences between some wild species are not obvious, and there are also some transitional species, which make the classification status difficult to reveal. 3) Finally, the difference selection of molecular marker often causes different analysis results of the evolutionary relationship in *Fagopyrum*. On the other hand, combined evidence from morphology and molecular biology has been used in many research aspects such as hybrid studies [36], phylogenetic study [15,37] and identification of new species [14,38]. 

Faced with abundant resources and complex evolutionary issues, we used different DNA barcodes which came from the comparative analysis of buckwheat species based on complete chloroplast genomes and combined them with morphology characteristics; meanwhile, we also collected 25 species from *Fagopyrum* including 2 cultivated species, 21 wild species and 2 variations, which is the most comprehensive species for phylogenetic research of buckwheat, in the end, we revealed the relationship between buckwheat and proved the *psbE-psbL* and *ndhA* intron were promising DNA barcodes for *Fagopyrum*, which could reflect the high base substitutions between wild and cultivated species and also resolved the issues of germplasm resources identification. Our research has developed the theory of two groups, we believe the *Fagopyrum* should be divided into a cultivated group and wild group, and the cultivated group (cymosum group) should include *F. tataricum, F. esculentum* and *F. cymosum*, as well as their relieved wild species which were distributed in wild environment and not cultivated widely, such as *F. tataricum* (sichuan) and *F. megaspartanum*. One the other hand, the wild group (urophyllum group) should have constituted most wild buckwheat like *F. jinshaense, F. longzhoushanense, F. urophyllum*, *F. leptopodum*; *F. gracilipes* and so on. At the same time, *F. cymosum* was the most widely distributed wild buckwheat species due to its strong adaptability of environments [39], which induced lots of synonym names of this species like *F. megaspartanium*. In this study, we compared the differences between *F. cymosum* and *F. megaspartanium*, and the results indicated the *F. megaspartanium* should be treated as *F. cymosum* based on the phylogenetic analysis and morphological characteristics.

Consequently, the phylogenetic analysis based on molecular markers shows great advantages in *Fagopyrum*, which could reveal the genetic divergence in sequence information of different species, and could also identify the new species clearly, and the *psbE-psbL* and *ndhA* intron were promising plastid DNA barcodes of buckwheat phylogenetic research. We believe our results could provide useful reference for fulfilling DNA barcodes of *Fagopyrum* taxonomy research and wild buckwheat species identification in the future.

## 4. Materials and Methods 

### 4.1. Plant Populations

In this study, 25 buckwheat species were used for the morphological and phylogenetic research, including 2 cultivated species, 21 wild species and 2 variants. The wild buckwheat populations were collected during wild buckwheat investigation from 2015 to 2018 in Southwest China. The detail and collected places of different materials are shown in Table 1.

### 4.2. Observation and Analysis of Morphological Characteristics

The two months old seedlings of different species were selected for morphological observation. In this research, we mainly focused on the height, leaf and fruit morphology of buckwheat, including plant height, leaves length, leaf width, seeds length and seeds width, as well as the karyotype and the genetic stability of species which was evaluated by self-sterility or not. The diploid and tetraploid were represented by 2 and 4 respectively, meanwhile, the self-sterility and self-infertility were represented by 1 and 2 when we analyzed these data. All measurement data were designed in completely randomized block design and calculated for three individual plant of each species. Additionally, the Principal Component Analysis (PCA) was processed using IBM SPSS statistics v24 (IBM co., New York, NY, USA) to reflect the differences among different species in *Fagopyrum* that could reduce the dimensionality of the morphology data as well as the karyotype information and so on. The scatter plot of different buckwheat was drawn using two component factors, which could reflect the differences between buckwheat species preliminarily, and the inter-species relationship in *Fagopyrum* will also be revealed.

### 4.3. Genome DNA Isolation and Molecular Barcodes Amplification

We used five different plastid DNA barcodes to demonstrate the phylogenetic relationships in *Fagopyrum*. In order to validate the promising molecular markers for the wild resources identification and phylogenetic research in the future, two widely used molecular barcodes *matK* [15] and *rbcL-accD* [18,19], and three intergenic regions of chloroplast DNA, including *ndhA* intron, *trnT-trnL* and *psbE-psbL*, which came from the results of comparative analysis based on chloroplast genomes [16], were selected in this research. 

The primers designed for PCR amplification are illustrated in Table S1. Meanwhile, the young leaves of buckwheat from individual seedlings were sampled for total genome DNA isolation using a plant genome extraction kit (TaKaRa co., Beijing, China). The sequences of different molecular barcodes were amplified separately, and the amplification was processed as follows: 95 °C for 4 min, 32 cycles of 95 °C for 30 s, 56 °C for 30 s and 72 °C for 60 s, and the final extension for 8 min at 72 °C. After that, the products were cloned into a pMD19-T (TaKaRa co.) vector and sequenced by ABI 377 DNA Sequencer (Thermo Scientific co., Beijing, China), and the doubtful bases were verified with a third sequencing reaction to avoid errors. The length of the *psbE-psbL* was about 1300 bp, the length of the *ndhA*-intron was about 1100 bp, and the length of the *trnT-trnL* was about 1000 bp, respectively. 

### 4.4. Phylogenetic Analysis

The phylogenetic analysis was performed based on different molecular marker sequences of *Fagopyrum,* and other taxon of dicotyledonous plants such as outgroup including four species *Rheum palmatum*, *Oxyria sinensis*, *Agrostemma githago* and *Salicornia bigelovii* came from Polygonaceae, Caryophyllaceae, and Chenopodiaceae, respectively, which could provide more information for the phylogenetic trees constriction, and its nucleotide sequence data were obtained from NCBI.

Subsequently, the sequences of different species were aligned by the CLC-Workbench using the blast program (CLC Bio Qiagen, Hilden, Germany). Individual gap positions were treated as missing data. Meanwhile, the sequences at both ends that came from cloning vectors were deleted. After that, the phylogenetic trees were inferred by three different methods including Maximum Likelihood (ML), Maximum Parsimony (MP) and Bayesian Inference (BI). Meanwhile, the phylogenetic trees based on the ML method were processed by MEGA 7.022 [40] as well as the bootstrap replicates were 1000. The phylogenetic trees which were based on the MP method were performed using PAUP v4.0b1023 [41], and the Heuristic search was set to 1000 random addition sequences. About the phylogenetic trees based on BI method was conducted using MrBayes v3.2.624 [42], with Markov chain Monte Carlo simulations run twice for 2 million generations independently; the phylogenetic trees were used to construct a majority-rule consensus tree after discarding the first 25% of trees.

Then, indeed, in order to further explore the evolutionary trends of different buckwheat species, which also confirms the potential role of *psbE-psbL* and *ndhA* intron in the phylogenetic study in *Fagopyrum*, five different combination of molecular markers were carried out in the construction of the phylogenetic trees, which combined signal molecular sequences together, including *matK* + *psbE-psbL*, *matK* + *ndhA* intron, *ndhA* intron + *psbE-psbL*, *rbcL-accD* + *psbE-psbL*, and *matK* + *ndhA* intron + *psbE-psbL*. It could offer more sequences information to verify the evolutionary relationship among buckwheat, as well as cover two high venation reigns which came from LSC and SSC in the chloroplast genome and two widely used DNA barcodes.

## Figures and Tables

**Table 1:** Materials and collecting places of *Fagopyrum* species used in this research.

Number	Category	Species	Locations
1	Cultivated species	*F. esculentum* cv. *T12*	Chengdu, Sichuan
2		*F. tataricum*	Chengdu, Sichuan
3	Wild species	*F. esculentum* ssp. *ancestralis*	Diqing state, Yunnan
4		*F. tataricum* (sichuan)	Aba state, Sichuan
5		*F. tataricum* (yunnan)	Yuxi city, Yunnan
6		*F.cymosum* (sichuan)	Liangshan state, Sichuan
7		*F.cymosum* (yunnan)	Dali state, Yunnan
8		*F. megaspartanum*	Diqing state, Yunnan
9		*F. pugense*	Liangshan state, Sichuan
10		*F. crispatifolium*	Liangshan state, Sichuan
11		*F. qiangcai*	Aba state, Sichuan
12		*F. wenchuanense*	Aba state, Sichuan
13		*F. luojishanense*	Liangshan state, Sichuan
14		*F. longzhousahnense*	Liangshan state, Sichuan
15		*F. gracilipes*	Dali state, Yunnan
16		*F. urophyllum*	Dali state, Yunnan
17		*F. leptopodum*	Yaan city, Sichuan
18		*F. jinshaense*	Lijiang city, Yunnan
19		*F. rubifolium*	Aba state, Sichuan
20		*F. callianthum*	Aba state, Sichuan
21		*F. capillatum*	Lijiang city, Yunnan
22		*F. lineare*	Dali state, Yunnan
23		*F. macrocarpum*	Aba state, Sichuan
24	Variation	*F. gracilipes* var. *odontopterum*	Lijiang city, Yunnan
25		*F. gracilipes* var*. odontopterum-R*	Liangshan state, Sichuan

Note: Differences between *F. gracilipes* var. *odontopterum-R* and *F. gracilipes* var. *odontopterum* is *F. gracilipes* var. *odontopterum-R* has red wing color while *F. gracilipes* var. *odontopterum* is white.

## Supplementary Materials

Supplementary materials can be found at https://www.mdpi.com/1422-0067/20/14/3455/s1.
