# Supplementary material for: psbE-psbL and ndhA Intron, the Promising Plastid DNA Barcode of Fagopyrum
Source: Int J Mol Sci. 2019 Jul 14;20(14):3455. doi: 10.3390/ijms20143455 (PMC6678665; doi:10.3390/ijms20143455)
Supplement: Supplementary file 1 [file ijms-20-03455-s001.zip › ijms-527597-supplementary-Final/Supplementary material 2-0712 Final.docx]

**Supplementary material 2**

**Supplementary Table 1 Primer designed for the phylogenetic analysis of *Fagopyrum***

| **DNA barcode** | **Forward (5’--3’)** | **Reverse (5’--3’)** |
| --- | --- | --- |
| *matK* | ATTTATGCACTTGTACATAAT | GAAGAATCTCTTGATAAGAT |
| *trnT-trnL* | ACAAAGATGCAATTCAGATCA | ATGGGACTCTATCTTCATTCT |
| *psbE-petL* | ATGCGCCAATTACTAAAAAGAG | TCCGAATTGTCCCTGAATCTCA |
| *rbcL-accD* | TGGTTGTGCTGGATCCACAAT | AATGAAGATAACTGTCAATA |
| *ndhA-intron* | AATTCCACCCCCATAAACCAT | TTTCCTATGTACAAGAATTAC |

**Supplementary Figure 1 The electrophoresis for PCR products for the *ndhA-intron***


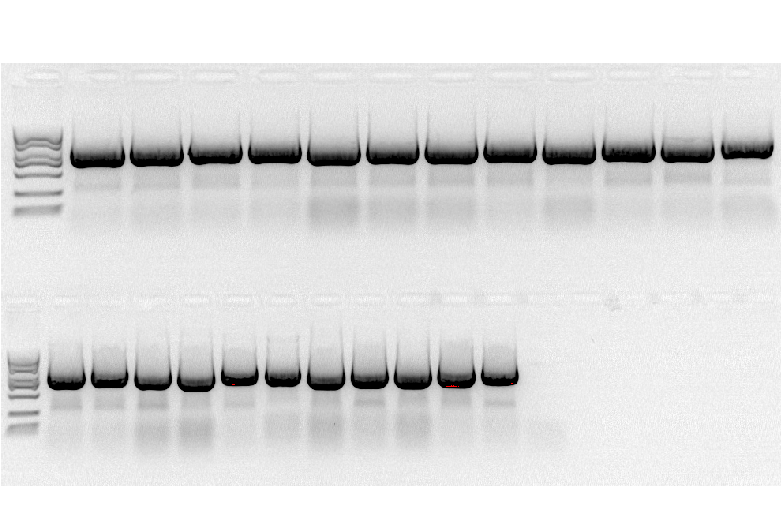


The electrophoresis for PCR products for the *ndhA-intron* was illustrated below. The first column of each line showed the DL2000 plus DNA Marker, and the marker represented 2000 bp, 1500 bp, 1000 bp, 750 bp, 500 bp, 250 bp and 100 bp from top to bottom. Meanwhile, the PCR products came from different *Fagopyrum* species, including *F. wenchuanense, F. esculentum* ssp. *ancestralis, F. gracilipes, F. gracilipes* var. *odontopterum, F. gracilipes* var. *odontopterum*-R*, F. leptopodum, F. crispatifolium, F. luojishanense, F. tataricum* (sichuan)*, F. macrocarpum, F. longzhoushanense, F. rubifolium, F. qiangcai, F. callianthum, F. jinshaense, F. capillatum, F. puge, F. megaspartanum, F. lineare, F. tataricum* (yunnan)*, F. cymosum* (yunnan)*, F. cymosum* (sichuan)*,* and *F. esculentum* cv. *T12* from left to right, respectively.
